# Vaccination rescues dysfunctional T cell therapy by amplifying rare stem-like antitumor CD8^+^ T cells

**DOI:** 10.21203/rs.3.rs-8941883/v1

**Published:** 2026-03-13

**Authors:** Sri Krishna, Zhiya Yu, Mohona Chakravarti, Victoria Dulemba, Aaron J. Dinerman, Lior Levy, Kyle J. Hitscherich, Jared J. Gartner, Sivasish Sindiri, Kristen Skonieczny, Todd D. Prickett, Paul F. Robbins, Stephanie L. Goff, Frank J. Lowery, James C. Yang, Steven A. Rosenberg

**Affiliations:** 1 Surgery Branch, Center for Cancer Research, National Cancer Institute, National Institutes of Health, Bethesda, MD 20892, USA.

## Abstract

Most antitumor CD8^+^ T cells in patients exhibit dysfunctional phenotypes, limiting the efficacy of adoptive cell transfer (ACT) against cancer. Although cancer vaccines can induce antitumor stem-like T cell (T_SL_) phenotypes, whether they can reverse T cell dysfunction during ACT remains unclear. Using murine neoantigen-specific tumor models, we show that concurrent neoantigen-vaccination enhances the antitumor activity of ACT-products dominated by dysfunctional T cells, relying on host antigen-presenting cells. Vaccination remodels the immunosuppressive tumor microenvironment and promotes the expansion of T_SL_ cells into tumors and lymphoid organs. Mechanistically, vaccination does not directly rescue dysfunctional T cells; but selectively amplifies low-frequency T_SL_ (as low as 0.1% in infusion) to mediate tumor control. Analysis of human dysfunctional TIL-ACT products containing scarce antitumor T_SL_ cells (~1%) administered to a patient with metastatic melanoma corroborated these findings, demonstrating complete clinical tumor regression, and expansion of adoptively transferred tumor-specific-TIL clonotypes only after concurrent vaccination. These data suggest that concurrent vaccines can unlock the therapeutic potential of rare stem-like T cells within otherwise ineffective dysfunctional ACT.

Adoptive cell transfer of tumor-infiltrating lymphocytes (TIL-ACT) has emerged as a promising therapeutic approach for patients with metastatic melanoma^1–5^ and other solid tumor malignancies^6,7^. TIL-ACT targeting somatic tumor mutations (neoantigens) offers the potential for highly specific tumor eradication with minimal off-target toxicity, making it a personalized cancer immunotherapy strategy^8^. Neoantigen-directed TIL-ACT can mediate complete regression of metastatic melanoma^8,9^ and other solid epithelial cancers traditionally considered immunologically “cold”^10–13^. Nevertheless, many patients with solid tumors frequently experience short-lived responses during TIL-ACT^5,10–14^. Thus, strategies that enhance the durability of ACT-mediated clinical tumor regressions in common solid epithelial tumors are of significant need.

Emerging evidence indicates that pre-dysfunctional, antitumor stem-like CD8^+^ T cells (T_SL_) are associated with complete responses to TIL-ACT^5,15^, and improved outcomes during immune checkpoint blockade (ICB) therapy^16^, chimeric antigen receptor (CAR) T cell therapies^17^, and T cell receptor engineered therapies (TCR-T)^18^. Stem-like antitumor T_SL_ cells can self-renew, differentiate into effector T cells, display low expression of cardinal T cell dysfunction markers such as CD39^5,15^, and mediate superior tumor control *in vivo*^15,19–24^. In contrast, chronic antigen stimulation drives dysfunctional T cells (T_dys_) within TIL to reduce expression of T_SL_ transcription factor *TCF7*^19–22,25^. Instead they display an increased expression of the lineage transcription factor *TOX*^26,27^, along with relatively higher cell surface protein expression of proteins PD-1, CD39, CD69, and TIGIT^15,28–31^.

Importantly, in human cancer patients, CD8^+^ antitumor T cells are often found enriched in dysfunctional, exhausted CD39^+^ T_dys_ compartments within the tumor microenvironment (TME), and consequently in adoptively transferred TIL infusion products^13,15,32–37^. Studies have shown that TIL-ACT infusion products derived from either melanoma or solid epithelial cancers are dominated by terminally differentiated CD8^+^ CD39^+^CD69^+^ T_dys_ cells with a very low proportion (<10%) of CD8^+^CD39^−^ CD69^−^ T_SL_^13,15,37^. *In vitro* expanded CD39^+^CD69^+^ T_dys_ cells exhibit impaired proliferative capacity, diminished cytokine production, and diminished antitumor response *in vivo* when compared to their stem-like counterparts^15,27,29^. Thus, in patients with advanced cancer, the low frequency of T_SL_ against pre-existing tumor antigens (or neoantigens) can undermine the effectiveness of immunotherapies^13,15^. To this end, overcoming the phenotypic dysfunction and augmenting persistence, function of adoptively transferred T cells remains a critical objective for improving ACT efficacy against many solid tumors.

Therapeutic vaccines can synergize with immunotherapies and are currently being explored in human clinical trials^21,38–42^. In murine studies, vaccination has been shown to induce *de novo* antitumor TIL with Tcf-1^+^ (*TCF7*) T_SL_ phenotypes to promote antitumor T cell responses in combination with immunotherapies such as ICB^21,43^. Murine models have also shown that concurrently administered vaccines enhance the antitumor effect of adoptively transferred T cells^44^, although the mechanisms behind vaccine-mediated enhancement of ICB and ACT particularly in the context of T_dys_ cells, is unclear. For instance, some studies have emphasized the role of T_SL_ in these models, while others have suggested that vaccines may directly reverse T cell dysfunction by converting suboptimally primed tumor antigen-specific T cells into properly primed T cells^45^.

In this study, we investigated the impact of therapeutic cancer vaccines administered along with ACT of phenotypically distinct T cell subsets. The Pmel TCR-transgenic murine model targeting a gp100 self-epitope has been instrumental in dissecting the determinants of successful ACT^46–48^. Recently, CD8^+^ Pmel T cells were demonstrated to also recognize a mutated gp100-neoepitope (gp100^KVP^) that is more immunogenic than the wildtype murine gp100 self-epitope^49^. These neoantigen-tumor models provide a unique opportunity to evaluate and understand vaccine-specific mechanisms and their impact on specific T cell subsets including T_SL_ and T_dys_ cells during ACT.

## Results

### Concurrent vaccination enhances the antitumor effect of cell therapy dominated by dysfunctional CD8^+^ T cells.

**Figure 1.**
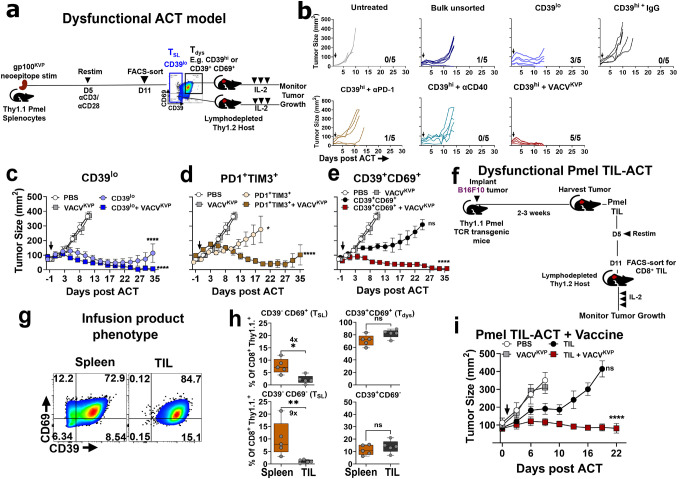
Concurrent vaccination enhances the antitumor effect of cell therapy composed of dysfunctional CD8^+^ T cells. **a.** Schema for generating Pmel ACT infusion composed of CD39^lo^ T_SL_ cells, or CD39^hi^, CD39^+^CD69^+^ T_dys_ cells targeting neoantigen expressing B16 melanoma or MC38 colon tumors (Extended Figs 2–3). **b.** Concurrent vaccination using VACV^KVP^ enhances ACT using FACS-sorted CD39^hi^ T cells while other immunologic agents do not; ACT using CD39^lo^ T_SL_ cells shown as comparison. Numbers indicate surviving mice at the end of the experiment **c-e.** Concurrent vaccination using VACV^KVP^ enhances the antitumor effect of adoptively transferred, FACS-sorted (c) CD39^lo^T_SL_ cells or dysfunctional (d) PD1^+^TIM3^+^, and (e) CD39^+^CD69^+^ Pmel T cells. **f.** Schema for generating TIL-ACT infusion composed of dysfunctional Pmel TIL targeting neoantigen expressing B16 tumors. **g**. Cell surface phenotypic analysis of CD39 and CD69 expression in CD8^+^ Pmel T cells derived from spleen or Pmel TIL from the one representative mouse. **h.** Summary of flow cytometry analyses of all four CD39/CD69 phenotypic subsets between splenic T cells and TIL from mice (n = 5 mice). **i.** Antitumor effect of adoptively transferred Pmel TIL as generated administered with or without concurrent VACV^KVP^ vaccination. All *in vivo* experiments were conducted with n=5–6 mice per group. Statistical analysis for *in vivo* experiments by One-way ANOVA with Tukey’s multiple comparisons test, compared to vaccination alone (VACV^KVP^) unless indicated. ns - not significant. * *P* < 0.05, ** *P* < 0.01, *** *P* < 0.001, **** *P* < 0.0001.

Adoptive cell transfer of CD8^+^ Pmel T cells can cause tumor regressions in large established vascularized tumors expressing this neoepitope when transferred as bulk T cells (B16^KVP^ melanomas and MC38^KVP^ colon cancers, **Extended Data Fig. 1**)^49^. We previously reported that ACT using CD8^+^CD39^−^ CD69^−^ T_SL_ cells isolated from *in vitro* expanded Pmel T cells can mediate superior tumor regression when compared to CD39^+^CD69^+^ Pmel T_dys_ cells without utilizing any vaccination^15^. We modeled ACT using dysfunctional T cells (T_dys_-ACT) by subjecting Pmel splenocytes to two rounds of strong *in vitro* stimulation, followed by fluorescence-activated cell sorting (FACS) for markers of terminal dysfunction (e.g. >95% CD39^hi^ or CD39^+^CD69^+^, Fig. 1a–b, **Extended Data Fig. 2**) and subsequent *in vitro* and *in vivo* ACT-evaluation against gp100^KVP^ neoepitope-expressing tumors (**Extended Data Fig. 3**). As expected, CD39^hi^ or CD39^+^CD69^+^ T_dys_ cells displayed limited tumor control whereas CD39^lo−^ expressing T_SL_ (which includes CD39^−^CD69^−^ and CD39^−^CD69^+^) displayed clear antitumor activity both *in vitro* and *in vivo* (**Extended Data Fig. 3**). Notably, while co-administration of anti-PD-1 or agonistic anti-CD40 antibody did not improve ACT of CD39^hi^ T_dys_ cells *in vivo*, concurrent systemic intravenous (i.v.) neoantigen vaccination using vaccinia virus encoding the gp100^KVP^ neoepitope (VACV^KVP^) elicited significant tumor regression to the levels of ACT using CD39^lo^ T_SL_ cells (Fig. 1b). Similarly, ACT administered using FACS-sorted T_dys_ cells expressing alternative dysfunctional markers (PD1^+^TIM3^+^ or CD39^+^CD69^+^) when co-administered with VACV^KVP^ also led to significantly better tumor control than transfer using T_dys_ cells alone, indicating that concurrent vaccination consistently improved tumor control during T_dys_-ACT, irrespective of the cell-surface markers associated with T_dys_ used for cell separation (Fig. 1c–e). Similar to the previous results seen with transfer of CD39^−^CD69^−^ T_SL_^15^, ACT using FACS-sorted CD39^lo^ T_SL_ alone achieved superior tumor control compared to T_dys_ cells alone, which was further enhanced by concurrent vaccination to achieve complete cures in the experiment (Fig. 1c, **Extended Data Fig.3**). We observed similar results in the gp100^KVP^-expressing MC38 colon cancer tumor model wherein the antitumor effect of CD39^+^CD69^+^ T_dys_-ACT was significantly enhanced by concurrent VACV^KVP^ vaccination (**Extended Data Fig.4**).

To mirror dysfunctional TIL-ACT administered to humans, we harvested implanted B16F10 tumors in Pmel-transgenic mice, expanded Pmel TIL by *in vitro* stimulation, and infused CD8^+^ Pmel TIL into congenic B16^KVP^ tumor-bearing hosts (Fig. 1f). Reflecting profound TME-induced T cell dysfunction, flow cytometric analysis demonstrated that Pmel TIL infusion products displayed a substantially lower proportion of CD39^−^ T_SL_ cells (up to 9-fold lower CD39^−^CD69^−^) compared to splenic T cells derived from the same mice, with comparable levels of T_dys_ cells (Fig. 1g–h). Despite this, concurrent VACV^KVP^ vaccination significantly enhanced the antitumor efficacy of dysfunctional Pmel TIL-ACT, recapitulating the results we observed with T_dys_-marker-selected ACT on splenocytes (Fig. 1i). Taken together these results reinforce that T_dys_ cells are ineffective in the neoantigen-ACT model and demonstrate that antigen-specific vaccination can restore the function of more phenotypically dysfunctional cell therapy, including TIL-ACT.

### Host antigen presentation governs vaccine-mediated rescue of T_dys_-ACT.

**Figure 2.**
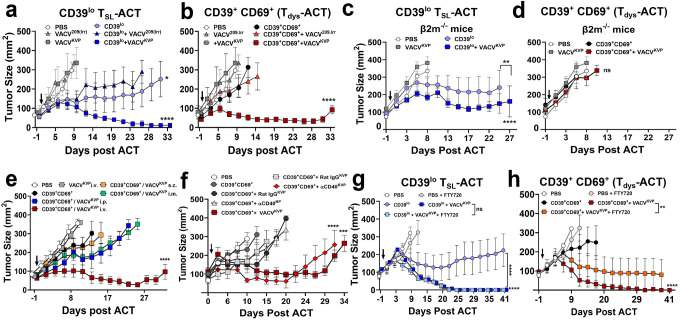
Host and vaccine modality parameters of enhancing stem-like and dysfunctional CD8^+^ T cells during ACT. **a-b.** Neoepitope in the vaccine backbone is required for enhancing ACT using dysfunctional T cells. *In vivo* antitumor effect of concurrent vaccination with cognate KVP epitope (VACV^KVP^) and irrelevant Pmel-epitope (VACV^209irr^) on ACT using (a) CD39^lo^ T_SL_ cells or (b) CD39^+^CD69^+^ T_dys_ cells. **c-d.** Host antigen presentation is needed for enhancing ACT using CD39^+^CD69^+^ T_dys_ cells by concurrent vaccine. *In vivo* antitumor effect of VACV^KVP^ vaccination during ACT using (c) CD39^lo^ T_SL_ ACT or (d) CD39^+^CD69^+^ T_dys_-ACT in β2m-deficient host. **e.** Impact of vaccine route in enhancing ACT using dysfunctional T cells. CD39^+^CD69^+^ T_dys_-ACT was performed along with concurrent VACV^KVP^ administered intravenously (i.v.), intraperitoneally (i.p.), subcutaneously (s.c.), or via intramuscular (i.m.) injection. **f.** *In vivo* antitumor effect using dysfunctional T cell ACT along with agonistic **α**CD40 antibody with KVP-neoepitope (**α**CD40^KVP^), or irrelevant Influenza epitope (**α**CD40^NP^) compared to control antibody with KVP-neoepitope (Rat IgG^KVP^) compared to VACV^KVP^. **g-h.** Effect of blocking lymphoid organ trafficking on vaccine-mediated enhancement of ACT using (g) CD39^lo^ T_SL_ ACT or (h) CD39^+^CD69^+^ T_dys_-ACT. Statistical analysis for *in vivo* experiments by One-way ANOVA with Tukey’s multiple comparisons test, compared to vaccination alone (VACV^KVP^) unless indicated. ns - not significant. * *P* < 0.05, ** *P* < 0.01, *** *P* < 0.001, **** *P* < 0.0001.

We sought to determine the factors that enable concurrent vaccination to enhance the antitumor activity of adoptively transferred T_dys_ and T_SL_ cells. We first evaluated the effects of concurrent recombinant vaccinia virus vaccination encoding either the cognate neoepitope (VACV^KVP^) or an irrelevant epitope (VACV^209irr^) on ACT using CD39^lo^ T_SL_ or CD39^+^CD69^+^ T_dys_ cells (Fig. 2a–b). CD39^lo^ T_SL_ cells alone delayed tumor progression and irrelevant VACV^209irr^ did not significantly enhance antitumor effect compared to VACV^KVP^ co-administration (Fig. 2a). In contrast, the antitumor effect of ACT using CD39^+^CD69^+^ T_dys_ cells required co-administration of the cognate neoepitope bearing VACV^KVP^ vaccine, while the irrelevant epitope VACV^209irr^ vaccine or cells alone completely lacked any antitumor effect (Fig. 2b).

To assess the importance of host antigen presenting cells, we performed ACT in β2-microglobulin-deficient (β2m^−**/**−^) mice, which lack MHC-I–mediated antigen presentation on host cells but not on implanted tumor cells (Fig. 2c–d). While ACT using CD39^lo^ T_SL_ mediated led to tumor regression in these host mice with or without concurrent vaccination (Fig. 2c) the ability of VACV^KVP^ to enhance ACT was completely abrogated for CD39^+^CD69^+^ T_dys_-ACT in β2m^−/−^ mice (Fig. 2d). An evaluation of multiple routes of vaccine delivery indicated that intravenous administration supported robust enhancement of T_dys_-ACT by VACV^KVP^, consistent with other reports (Fig. 2e)^43,50,51^. These results demonstrate that antigen specificity within the vaccine backbone, the vaccine route, and host antigen-presenting capacity together were indispensable for vaccine-mediated augmentation of dysfunctional T cell responses.

To evaluate if vaccinia virus-based vaccines had a unique ability to rescue dysfunctional T cells, we compared multiple vaccine modalities for their capacity to enhance T_dys_-ACT (Fig. 2f, **Extended Data Fig.5**). Systemic intravenous administration of recombinant αCD40 agonistic antibody admixed together with gp100^KVP^ synthetic peptide (αCD40^KVP^) significantly delayed tumor progression when combined with T_dys_-ACT, promoting efficacy comparable to that of VACV^KVP^ (Fig. 2f) whereas irrelevant or control vaccines (αCD40^NP^, Rat IgG^KVP^), or intraperitoneal αCD40^KVP^ had no impact and were no different from CD39^+^CD69^+^ T_dys_ cells alone (Fig. 2f, **Extended Data Fig.5a**). An adenovirus vaccine encoding the neoepitope (Adv^KVP^) also promoted significant tumor control when co-administered with T_dys_-ACT although the effect was short-lived (**Extended Data Fig.5b**). These data indicate that multiple vaccine modalities can mediate rescue of T_dys_-ACT with varying effectiveness.

Finally, to probe the role of secondary lymphoid organ trafficking, we blocked lymphocyte egress during therapy using the S1PR1-agonist FTY720^23^. While FTY720 treatment had minimal impact on ACT+vaccine with CD39^lo^ T_SL_ (Fig. 2g) it significantly impacted the therapeutic benefit of concurrent vaccination and T_dys_-ACT (Fig. 2h). Taken together, these results emphasize the impact of vaccine-antigen specificity, host antigen presentation, and lymphoid organ trafficking on the effectiveness of T_dys_-ACT. However, these parameters were less important for T_SL_-ACT presumably indicating the natural ability of less-dysfunctional T_SL_ cells to differentiate into effectors and mediate antitumor response.

### Concurrent vaccination reduces the immune-suppressive TME during T_dys_-ACT.

**Figure 3.**
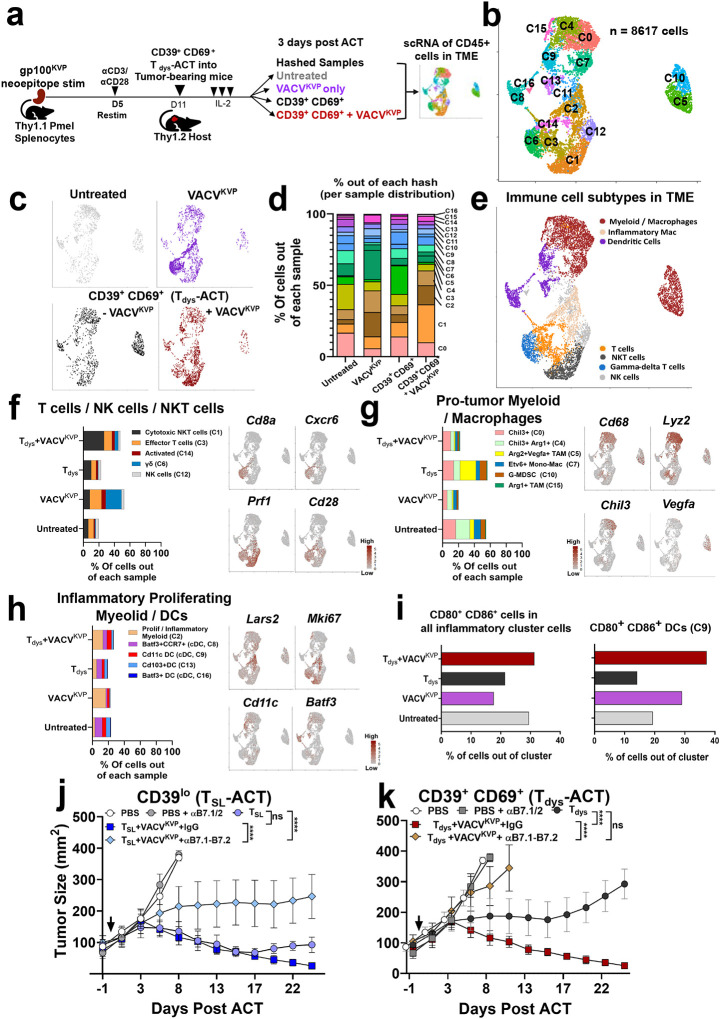
Concurrent vaccination reprograms the tumor microenvironment (TME) to alter the immune landscape during ACT using dysfunctional CD8^+^ T cells. **a.** Schema for analyzing vaccine mediated enhancement of ACT using CD39^+^CD69^+^ T_dys_ cells by scRNA-seq and deconvolution of hashed comparator groups. **b.** UMAP projection of single cell transcriptomes of all tumor-infiltrating immune cells from the 4 groups shown in a. **c-d.** Distribution of immune cells from each hashed group (c), and their quantification per each cluster (d). **e.** Broad immune subtype classification of clusters based on defined gene expression profiles and markers in the TME (see Extended Data Fig. 5). **f.** Proportion of T cells, NK cells, and NKT cells per each group (left) with select canonical genes projected on the UMAP (right). **g.** Proportion of pro-tumor myeloid, macrophage immune subtypes per each group (left), with select canonical genes projected on the UMAP (right). **h.** Proportion of antitumor inflammatory myeloid, DC immune subtypes per group (left) with select canonical genes projected on the UMAP (right). **i.** Proportion of CD80^+^CD86^+^ immune cells per each group within inflammatory myeloid cells, and Cd11c+ DCs (Cluster C9) showing VACV^KVP^ drives an increase in these immune subtypes in the TME. **j-k.** Blocking B7.1/B7.2 costimulation on host cells affects VACV^KVP^-mediated enhancement of on ACT using (j) CD39^lo^ T_SL_ cells or (k) CD39^+^CD69^+^ T_dys_ cells. Statistical analysis for *in vivo* experiments by One--way ANOVA with Tukey’s multiple comparisons test, ns - not significant, **** *P* < 0.0001.

We sought to understand if concurrent vaccination influences early changes in the immune landscape within B16^KVP^ TME following T_dys_-ACT into lymphodepleted hosts that led to improved antitumor response. To this end, 3 days after ACT, we performed single-cell RNA sequencing (scRNA-seq) along with cellular indexing of transcriptomes and epitopes by sequencing (CITE-seq) by “cell hashing” of 4 groups of CD45^+^ tumor-infiltrating immune cells^52^: those derived from untreated mice, mice receiving VACV^KVP^ only, and mice receiving T_dys_-ACT with and without vaccination (Fig. 3a, **Extended Data Fig.6**). Unsupervised dimensionality reduction by uniform manifold approximation and projection (UMAP) analysis of 8,617 single-cell transcriptomes across the 4 experimental conditions revealed distinct clustering of immune populations (18 clusters) within the TME (Fig. 3b, **Extended Data Fig.6, Supplementary Table 1**).

Demultiplexing of hashed samples enabled the immune cell deconvolution and quantification of tumor immune landscapes derived from the study groups (Fig. 3c–d). Canonical marker analyses identified the major immune cell lineages, including T cells (clusters 3 and 14), NK cells (cluster 12), NKT cells (cluster 1), γδ T cells (cluster 6), a significant proportion of myeloid/macrophage subsets (clusters 0, 2, 4, 5, 7, 10, and 15), and dendritic cells (DCs, clusters 8, 9, 13, 16) (Fig. 3b–e, **Extended Data Fig. 6, Supplementary Table 1**).

We observed that TME within groups receiving VACV^KVP^ showed an increased proportion of lymphocytes (~2.5-fold) including NKT cells, γδ T cells, and tumor-resident effector T cells relative to untreated or T_dys_-ACT without vaccination (Fig. 3f). In contrast, previously described anti-inflammatory pro-tumor-associated myeloid (TAM) lineage population^43,53^, characterized by high expression of *Cd68*, *Chil3*, *Arg1*, *Arg2*, and *Vegfa* within the TME decreased (~3-fold) in response to vaccination (with or without T_dys_-ACT) relative to unvaccinated groups, (Fig. 3g). Additionally, the pro-inflammatory, proliferative myeloid subset (cluster 2) marked by *Cdk8*, *Mki67*, *Lars2*, *Gm26197* increased in response to vaccination (Fig. 3h)^54^. However, we did not observe a consistent increase in all pro-inflammatory antigen presenting cell types. For instance, we did not capture tumor-resident B cells likely due to lymphodepletion (Fig. 3e), nor did we observe vaccine-induced increase in previously reported *Batf3+ Ccr7+* Dendritic cells (DCs) reported as being important for antitumor effector T cells during ACT (Fig. 3h)^55^. Nevertheless, we did observe a two-fold increase in *Cd11c+* DCs (cluster 9) in the TME of mice receiving T_dys_-ACT+VACV^KVP^ relative to T_dys_-ACT (Fig. 3h). We also observed an increase in the abundance of inflammatory DCs co-expressing the co-stimulatory molecules CD80 and CD86, particularly within cluster C9 (~3-fold) (Fig. 3i).

Prior murine studies have indicated the importance of Cd28-dependent costimulation in rescuing exhausted T cells during anti-PD-1 immunotherapy^56–58^. These studies, along with our data supporting the the presence Cd28^+^ TIL in the TME (Fig. 3f), and the importance of host antigen presentation for T_dys_-ACT+VACV^KVP^ (Fig. 2, Fig. 3h–i), prompted us to perform ACT+vaccine using T_SL_ and T_dys_ cells in the presence of B7.1/B7.2 blockade to interfere with host APC-mediated costimulation^57^ (Fig. 3j–k). B7.1/B7.2 blockade completely abrogated the therapeutic benefit of concurrent VACV^KVP^ during T_dys_-ACT (Fig. 3k), while CD39^lo^ T_SL_ still retained some therapeutic benefit, despite lack of strong host-mediated co-stimulation (Fig. 3j). Together, these data demonstrate that concurrent vaccination fundamentally reprograms the TME, reducing immunosuppression and enhancing lymphocyte trafficking and antigen presentation, and that these effects are likely essential for rescuing the antitumor activity of otherwise ineffective T_dys_-ACT.

### Dynamics and phenotypes of adoptively transferred T cell subsets after concurrent vaccination.

**Figure 4.**
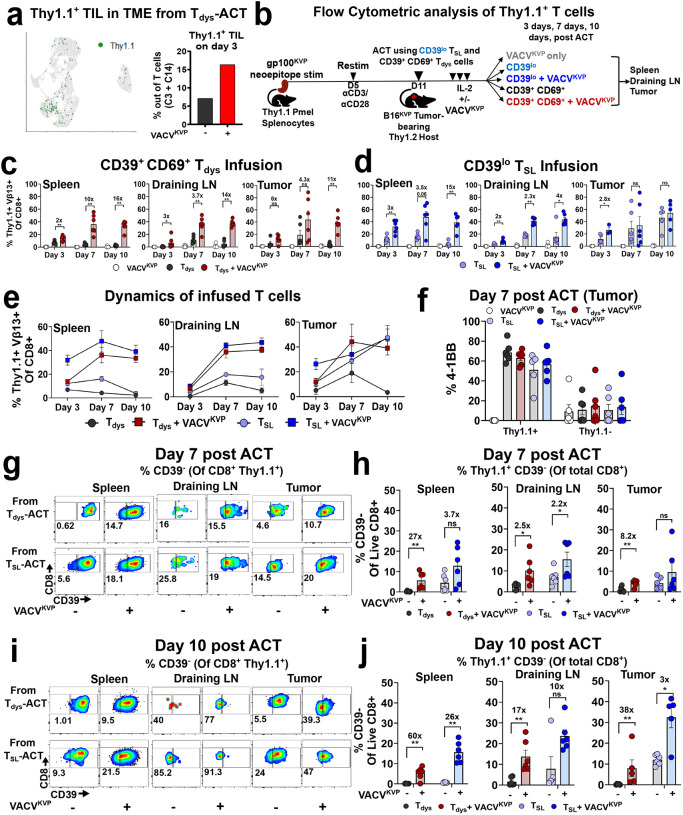
Dynamics and phenotype of adoptively transferred CD39^lo^ T_SL_ and CD39^+^ CD69^+^ T_dys_ cells after concurrent vaccination. **a.** UMAP showing distribution and proportion of adoptively transferred Thy1.1^+^ CD8^+^ T cells in the scRNA of TME (shown in Fig. 3) on the left (analyzed by Thy1.1^+^ CITESeq+ T cells) and quantification on the right. **b.** Schema for longitudinally analyzing frequency and phenotypes of adoptively transferred CD39^lo^ T_SL_ cells, and CD39^+^CD69^+^ T_dys_ cells with, or without concurrent vaccination from spleen, draining lymph nodes (DLN) and TIL (tumor). **c-d.** Frequency of adoptively transferred Pmel T cells derived from CD39^lo^ T_SL_-ACT, or CD39^+^CD69^+^ T_dys_-ACT across the 3 compartments. Numbers indicate fold change relative to counterpart unvaccinated groups from spleen, DLN, and tumor. Data points represent each mouse, n = 6 mice. **e.** Dynamics of adoptively transferred T cells over days 3, 7, and 10 across the 3 compartments +/− concurrent vaccination. Error bars indicate standard error of the mean. **f.** 4–1BB activation (tumor-recognition) of adoptively transferred (Thy1.1^+^), relative to irrelevant T cells (Thy1.1^−^) from CD39^lo^ T_SL_-ACT, or CD39^+^CD69^+^ T_dys_-ACT +/− concurrent vaccination within the TME at day 7 post ACT. Data points represent each mouse, n = 6 mice. **g-h.** Frequency of stem-like Thy1.1^+^ CD39^−^ T cells at day 7 post-ACT across 3 sites from adoptively transferred CD39^+^CD69^+^ T_dys_-ACT (top panel), and CD39^lo^ T_SL_-ACT (bottom panel). Representative flow cytometry plots with % of CD39^−^ T_SL_ cells out of Thy1.1^+^ CD8^+^ T cells indicated in (g), quantification out of all CD8^+^ T cells shown in (h). **i-j.** Frequency of stem-like Thy1.1^+^ CD39^−^ T cells at day 10 post-ACT across 3 sites from adoptively transferred CD39^+^CD69^+^ T_dys_ cells (top panel), and CD39^lo^ T_SL_ cells (bottom panel). Representative flow cytometry plots with % of CD39^−^ T_SL_ cells out of Thy1.1^+^ CD8^+^ T cells indicated in (i), quantification out of all CD8^+^ T cells shown in (j). Numbers indicate fold change relative to counterpart unvaccinated groups from spleen, DLN, and tumor. Data points represent each mouse, n = 6 mice. Statistical significance by unpaired Mann-Whitney test. ns - not significant. * *P* < 0.05, ** *P* < 0.01, *** *P* < 0.001, **** *P* < 0.0001.

The 3-fold increase in adoptively transferred Thy1.1^+^ Pmel T cells observed in the T_dys_-ACT+VACV^KVP^ group relative to T_dys_-ACT alone in the scRNA-seq data (Fig. 4a), prompted us to evaluate the impact of concurrent vaccination on specific T cell subsets. To this end, we phenotyped the adoptively transferred T_dys_ and T_SL_ cells with or without concurrent vaccination at 3-, 7-, and 10-days post-ACT in host tumor, spleen and draining lymph nodes (dLN) (Fig. 4b). Across the sites at all three timepoints, concurrent vaccination consistently increased Thy1.1^+^ T cells from both T_SL_ and T_dys_-ACT with the exception of cells from T_SL_-ACT in the tumor on days 7, and 10 (Fig. 4c–d). Notably, by day 10 post-ACT, concurrent vaccination resulted in over a 10-fold increase in the proportion of Thy1.1^+^ T cells in mice receiving T_dys_ in all 3 sites (Fig. 4c–d).

Longitudinal tracking indicated peak of transferred T cells on day 7 in the 3 sites consistent with previous reports^59^ (Fig. 4e). Even in the absence of vaccination, T_SL-_ACT cells were detectable in all three sites and continued to proliferate and increase within the dLN and tumor by days 7 and 10. In contrast, in the absence of vaccine, T_dys_-ACT cells proliferated the least with a continued decline and were barely detectable in all 3 sites by day 10 (Fig. 4e). Importantly, vaccination in both T_SL_ and T_dys_ cells resulted in sustained and dramatic proliferation of Thy1.1^+^ T cells (between 40–60% of all CD8^+^ T cells) that continued rising on days 7 and 10 in spleen, dLN, and tumor (Fig. 4e). Most transferred intratumoral Thy1.1^+^ cells were neoantigen-activated since they expressed the activation marker 4–1BB (>50%) compared to the host Thy1.1^−^ subset (<5%), with no significant differences between T_SL_ and T_dys_ ACT groups (Fig. 4f).

To evaluate the phenotypes of adoptively transferred T cells, we analyzed post-ACT phenotypes of Thy1.1^+^ cells in the spleen, dLN, and tumor on days 7 and 10 at the peak of T cell expansion (Fig. 4g–j). Consistent with previous reports including ours^15,19,60^, we detected self-renewed Thy1.1^+^CD39^−^ T_SL_ derived from CD39^lo^ T_SL_-ACT in all 3 physiologic compartments on day 7 which was further enhanced by vaccination (bottom panel in Fig. 4g, between 2–3 fold out of CD8^+^ cells in Fig. 4h). By day 10, Thy1.1^+^CD39^−^ T_SL_ cells derived from CD39^lo^ T_SL_-ACT displayed further expansion in all 3 sites (bottom panel of Fig. 4i, between 3–26-fold out of CD8^+^ T cells in Fig. 4j). In contrast, without vaccination, at days 7 and 10 post-ACT using CD39^+^CD69^+^ T_dys_ cells, we observed very little Thy1.1^+^CD39^−^ T_SL_ derived from CD39^+^CD69^+^ T_dys_-ACT across all sites (between 0.1–3% of CD8^+^ T cells in Fig. 4h, 4j) confirming poor persistence and expansion of terminally differentiated dysfunctional T cells as previously reported^15,21,23,27,30,61^. Despite this, concurrent vaccination resulted in a substantial increase of Thy1.1^+^CD39^−^ T_SL_ from adoptively transferred CD39^+^CD69^+^ T_dys_ cells in all 3 physiologic compartments at both day 7 (top panel in Fig. 4g, between 2.5–27-fold out of CD8^+^ T cells in Fig. 4h), and on day 10 after ACT (top panel in Fig. 4i, between 17–60-fold out of CD8^+^ T cells in Fig. 4j). These studies suggest that concurrent neoantigen vaccination may modulate and impact the frequency and phenotype of adoptively transferred T cells by increasing the proportion of T_SL_ cells *in vivo* to mediate antitumor immunity.

### Vaccination selectively expands low-frequency CD8^+^ T_SL_ cells within T_dys_ infusion products to enhance antitumor immunity.

**Figure 5.**
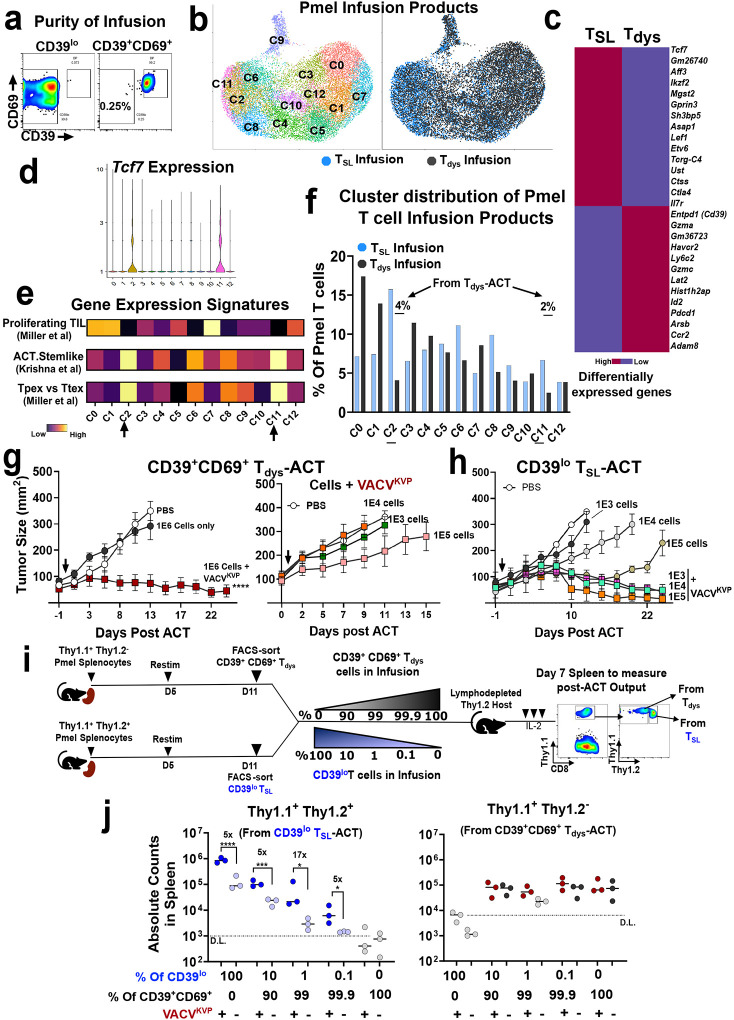
Concurrent vaccination amplifies low-frequency stem-like CD8^+^ T cells within dysfunctional T cell infusion products to enhance antitumor response. **a.** Representative purity of FACS-sorted CD39^lo^ and CD39^+^CD69^+^ T cell infusion products administered in *in vivo* studies. **b.** UMAP projection of single cell transcriptomes of FACS-sorted CD39^lo^ and CD39^+^CD69^+^ T cell infusion products; left - all cells, right - cells highlighted from CD39^lo^ T_SL_ infusion (blue), and CD39^+^CD69^+^ T_dys_ infusion (black). **c.** Top 20 differentially expressed genes between CD39^lo^ T_SL_ and CD39^+^CD69^+^ T_dys_ infusion products. **d.** Violin plot showing *Tcf7* expression in clusters 2 and 11. **e.** Single cell gene-set enrichment analysis (scGSEA) showing clusters 2 and 11 are enriched with ACT stem-like (ref 15)^15^, and Tpex (ref 20)^20^ T cell signatures. **f.** Distribution of T cells derived from CD39^lo^ T_SL_ and CD39^+^CD69^+^ T_dys_ infusion products in the UMAP clusters. Arrows and numbers indicate the % of T cells derived from CD39^+^CD69^+^ T_dys_ cell infusions within clusters 2 and 11. **g.** Antitumor effect of adoptively transferred CD39^+^CD69^+^ Pmel T cells +/− VACV^KVP^ vaccination (1E6 cells, left). and at lower cell numbers (right). **h.** Antitumor effect of low numbers of adoptively transferred CD39^lo^ T_SL_ cells +/− VACV^KVP^ vaccination. **i.** Schema for analyzing frequency of very low proportion of adoptively transferred CD39^lo^ Thy1.1^+^Thy1.2^+^ T_SL_ cells spiked into CD39^+^CD69^+^ Thy1.1^+^Thy1.2^−^ T_dys_ cells +/− VACV^KVP^ vaccination at 7 days post-ACT in the spleen. **j.** Quantification of recovered absolute numbers of CD39^lo^ T_SL_ cells from host spleen (light blue – no vaccine, dark blue – with vaccine) spiked into CD39^+^CD69^+^ T_dys_ cells (black- no vaccine, dark red – with vaccine), with proportions indicated below the plot (left); absolute numbers of adoptively transferred CD39^+^CD69^+^ T_dys_ cells (right). Light grey dots refer to baseline recipient population with no spike in corresponding to detection limit (D.L) (i.e. 100% T_dys_ cells for left and 100% T_SL_ cells for right plots respectively). Statistical significance by unpaired Mann-Whitney test. Numbers indicate fold change relative to counterpart unvaccinated groups. Statistical analysis for *in vivo* experiments by One--way ANOVA with Tukey’s multiple comparisons test, ns - not significant. * *P* < 0.05, ** *P* < 0.01, *** *P* < 0.001, **** *P* < 0.0001.

The unexpected and sustained expansion of CD39^−^ T_SL_ *in vivo* in mice treated with terminally differentiated CD39^+^CD69^+^ T_dys_-ACT suggested that vaccination was either: **a)** rescuing the phenotypes by amplifying terminally-differentiated T_dys_ cells perhaps by providing the appropriate APC-stimulation suggested previously^45^, or **b)** dramatically amplifying very low frequency T_SL_ “impurity” in our FACS-sorted T_dys_-ACT infusion product. To this end, we further reexamined the phenotypes of adoptively transferred CD39^lo^ T_SL_ and CD39^+^CD69^+^ T_dys_ cell infusion products (Fig. 5).

T_SL_ cell impurity in our FACS-sorted CD39^+^CD69^+^ T_dys_-ACT infusion is generally <1% (representative sort in Fig. 5a). Granular analysis of phenotypic states derived from a combined scRNA-seq of CD39^lo^ T_SL_ and CD39^+^CD69^+^ T_dys_ ACT-infusion products obtained immediately after FACS-sorting indicated that CD39^lo^ T_SL_ cells had a relatively higher gene expression of *Tcf7*, *Lef1*, *Ltb*, and *Il7r*, and a lower expression of T cell effector and dysfunction genes such as *Gzma*, *Gzmb*, *Havcr2, Pdcd1*, and *Entpd1*, consistent with previous reports in human TIL-ACT products (Fig. 5b–c, **Extended Data Fig. 7, Supplementary Table 2**)^15^. Focused analysis of *Tcf7* expression indicated that clusters 2 and 11 represented Tcf7^hi^ expressing stem-like phenotypic states (Fig. 5d), which was also reflected using single-cell gene expression analysis using signatures derived from stem-like human TIL^15^, and murine progenitor-exhausted T cells (Tpex) T cells^20^ (Fig. 5e). CD39^lo^ T_SL_-ACT derived cells largely predominated within clusters C2, C6, C8, and C11, with minor distribution of cells in other transcriptomic clusters (Fig. 5b, 5f). Importantly, clusters 2 and 11 contained approximately 4% and 2.2% of T cells derived from CD39^+^CD69^+^ FACS-sorted T cells respectively, demonstrating that the T_dys_-ACT infusion contained a small but detectable T_SL_ “impurity” (up to 6.2%) in our ACT infusion products (Fig. 5b, 5f).

To interrogate if small proportions of T_SL_ within CD39^+^CD69^+^ T_dys_ cell infusion products were being impacted by concurrent vaccination, we first evaluated the dosage effect of vaccine+ACT using T_dys_ and T_SL_ cells (Fig. 5g–h). Functionally, concurrent vaccination failed to rescue T_dys_-ACT at cell doses lower than 1×10^6^ CD39^+^CD69^+^ T_dys_ ACT, (Fig. 5g). By contrast, transfer of low numbers of stem-like CD39^lo^ T_SL_ (as few as 1,000 cells) along with concurrent VACV^KVP^ vaccination resulted in dramatic antitumor responses (comparable to 1×10^6^ CD39^lo^ T_SL_ used in prior experiments), even as the cells alone in the absence of vaccination in such small numbers were unable to sustain the same antitumor effect (Fig. 5h).

We then sought to directly assess whether vaccination mediated the expansion of rare stem-like T cells within a predominantly dysfunctional T cell infusion product. To this end, we co-transferred controlled, traceable populations of CD39^lo^ T_SL_ (ranging from 0.1–10%) expanded from Thy1.1^+^Thy1.2^+^ Pmel splenocytes into a background of CD39^+^CD69^+^ T_dys_ cells (ranging from 90–99.9%) expanded from Thy1.1^+^Thy1.2^−^ mice and quantified their frequency in the spleen seven days after adoptive cell transfer (ACT), with or without concurrent vaccination (Fig. 5i). We also included CD39^lo^ T_SL_ alone, or CD39^+^CD69^+^ T_dys_ cells without any admixed cells as controls. Strikingly, even at 0.1% and 1% frequencies of T_SL_ admixture, vaccination led to a significant increase (between 5–17-fold, *P* < 0.01, Mann-Whitney test) in the absolute number of the adoptively transferred Thy1.1^+^Thy1.2^+^ CD39^lo^ T_SL_ cells in the spleen, while the numbers of dysfunctional Thy1.1^+^Thy1.2^−^ CD39^+^CD69^+^ T_dys_ cells were unaffected and remained stable regardless of vaccination (Fig. 5j). These findings directly demonstrate that concurrent vaccination can selectively amplify rare, stem-like CD8^+^ T cells within a largely dysfunctional infusion product to mediate antitumor responses, but does not rescue or reverse the poor proliferation, phenotypic dysfunction of T_dys_ cells *in vivo* in our experiments.

### Concurrent vaccination amplifies low-frequency antitumor T cells within a dysfunctional TIL infusion product administered to a patient with metastatic melanoma.

**Figure 6.**
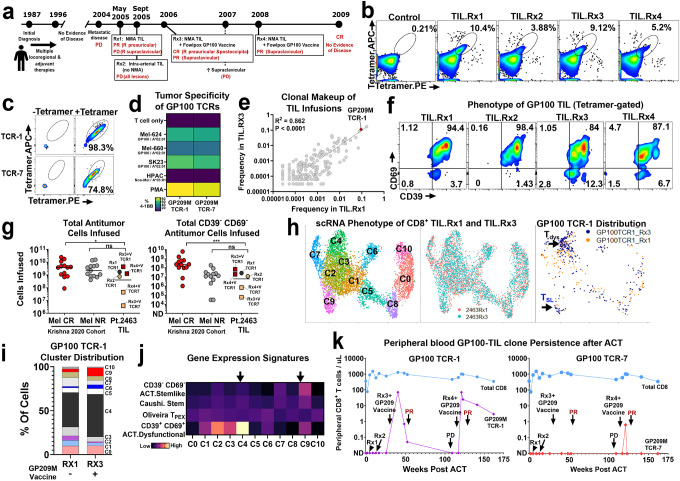
Concurrent vaccination amplifies low-frequency antitumor T cells within a dysfunctional TIL-infusion product administered to a metastatic melanoma patient. **a.** Clinical course of patient with metastatic melanoma treated with 4 subsequent TIL infusion products, reported in ref 62^62^. TIL infusions Rx3 and Rx4 were administered along with a Fowlpox gp100 vaccine (+V). PR - Partial response, PD - Progressive disease, CR - Complete response. NED- No evidence of disease. **b.** Frequency of gp100-specific tetramer+ TIL within the 4 TIL infusion products. Numbers indicate % out of live CD8^+^ TIL. **c.**gp100-reactive TCRs isolated from the 4 TIL infusion products showing specificity to tetramer. **d.** gp100-reactive TCRs isolated from TIL infusion showing tumor-specificity to A*02:01+ gp100-expressing melanoma tumors but not A*03:01+ non-melanoma line (HPAC). Numbers indicate % 4–1BB (activation) out of live CD8^+^ TCR-transduced T cells. **e.** CDR3β sequencing between TIL infusions Rx1 and Rx3 (+V). TCR-1 recognizing gp100 is shown. TCR-7 was below detection limit. **f.** Cell surface phenotypic analysis of CD39 and CD69 expression within CD8^+^ gp100-specific tetramer+ TIL within the 4 TIL infusion products. **g.** Absolute numbers of (left) total gp100-specific TIL infused to the melanoma patient, compared to neoantigen-specific TIL administered as ACT to complete responders (CR) and non-responders (NR) described in ref 15^15^; (right) stem-like CD39^−^ CD69^−^ antitumor TIL administered in the same melanoma cohort. Statistical significance by unpaired Mann-Whitney test. ns - not significant. * *P* < 0.05, *** *P* < 0.001. **h.** UMAP projection of all single cell transcriptomes of TIL-infusions (left) showing cells derived from Rx1 and Rx3 (middle); (right) distribution of gp100 TCR-1 clonotype on the UMAP is shown in Rx1 (orange) and Rx3 (dark blue). TCR-7 was below detection limit. **i.** Cluster distribution of gp100 TCR-1 clonotype in Rx1 and Rx3(+V). **j.** scGSEA gene-expression signature analysis indicating cluster C9 as a stem-like state and C4 as dysfunctional CD39^+^CD69^+^ state. **k.** Absolute counts of the 2 antitumor TCR clonotypes gp100 TCR-1 and TCR-7 in pre-, and post-ACT PBL of patient. Total CD8s are indicated in blue. Treatment courses and clinical responses are noted by arrows.

To evaluate whether concurrent vaccination can rescue dysfunctional cell therapy in humans, we analyzed the phenotypic states and clonal dynamics of TIL-ACT administered to a patient with metastatic melanoma at the Surgery Branch, the only published report of TIL-ACT along with antigen-specific vaccination^62^ (Fig. 6). The patient experienced a short-lived mixed response and developed progressive disease after conventional intravenous TIL-ACT (Rx1), and intra-arterial TIL infusion (Rx2), both targeting the HLA-A*02:01-restricted gp100 melanoma tumor antigen (GP209M epitope, Fig. 6a)^62^. Subsequently, the patient was treated with the intravenous TIL-ACT (similar to Rx1) along with co-administration of a Fowlpox vaccine encoding a GP209M-epitope (gp100 vaccine, Rx3+V) resulting in a remarkable regression of multiple lesions ultimately achieving a durable complete response of remaining lesions after second round of TIL+vaccination (Rx4+V) (Fig. 6a)^62^.

Because the administered TIL was polyclonal, it is conceivable that vaccine-induced gp100-reactive TIL clones with altered stem-like phenotypes initiated durable clinical antitumor response after TIL-ACT (Rx3 and Rx4) compared to those administered originally with minimal response (Rx1 and Rx2). To investigate this issue, we performed deep clonotypic and phenotypic analysis of the four TIL-ACT products administered to the patient. HLA-A*02:01-GP209M epitope-specific tetramer staining estimated a small proportion of antitumor T cells within the TIL-ACT (between 4–10% of CD8^+^ TIL)^62^ (Fig. 6b). Tetramer-based TCR isolation from all the 4 TIL infusions identified two gp100-specific TCRs (TCR-1 and TCR-7, see methods) with antigen-specific recognition of HLA-matched melanoma tumors *in vitro* (Fig. 6c–d). No additional new gp100-specific TCRs were detected from post-ACT peripheral blood lymphocytes (PBL).

CDR3β-based deep TCR-sequencing of TIL infusion products indicated high concordance of the adoptively transferred TCR-clonotypes between Rx1, Rx2, and Rx3 indicating that clonotypic makeup of TIL-ACT products had not significantly changed between infusions (Fig. 6e, **Extended Data Fig. 8**). In the final TIL infusion (Rx4), the inclusion of additional gp100-targeting *in vitro* expanded TIL fragments to the infusion product resulted in slightly altered clonal makeup, as expected (**Extended Data Fig. 8**). Nevertheless, the TCR-1 clonotype appeared immunodominant, with comparable frequencies in all four TIL infusion products (~5–10% of CD8^+^ T cells) while TCR-7 was sub-dominant and low in frequency or sub-detection limit in the TIL infusions (Fig. 6b–e, **Extended Data Fig. 8–9**). These concordant tetramer and TCR-sequencing results indicated that clinical responses observed in Rx3 and Rx4 along with concurrent vaccination were likely due to a single dominant gp100 TIL clonotype (TCR-1) in the administered TIL, and not due to heterogeneity of transferred TIL clones at the time of vaccination.

Flow cytometry-based tetramer phenotyping indicated that gp100-specific TIL were highly differentiated and dysfunctional (>84% CD39^+^CD69^+^ T_dys_ cells) with < 3% CD39^−^CD69^−^ T_SL_ in all four infusion products (Fig. 6f). We next compared the absolute number of infused gp100 TCR-1 and TCR-7 cells (total and T_SL_ cells) in the four different TIL infusions to the absolute number of tetramer-positive antitumor TIL published in our prior melanoma TIL-ACT cohort^5,15^. Importantly, we observed that stem-like gp100-TIL clones infused in this patient across the four TIL infusions were significantly lower than those administered to complete responders to TIL-ACT in our previously reported melanoma cohort^15^ (*P* < 0.001, Mann-Whitney test, Fig. 6g).

Higher resolution paired scRNA-seq and single-cell TCR sequencing analyses on CD8^+^ TIL from Rx1 (no vaccine) and Rx3 (with vaccine) infusions indicated that TIL cluster 4 mapped to terminally differentiated CD39^+^CD69^+^ T_dys_ cells, clusters 0 and 10 to proliferating Ki67+ TIL, and cluster 9 to T_SL_-like states (Fig. 6h–j, **Extended Data Fig. 9, Supplementary Table 3**). The immunodominant gp100 TCR-1 TIL-clonotype was distributed evenly across both CD8^+^ TIL within the infusions, with the majority of them in the cluster 4 (Rx1 = 40%, Rx3 = 48.3%), followed by cluster 0 (Rx1 = 9.9%, Rx3 = 10.2%), and cluster 3 (Rx1 = 3.6%, Rx3 = 9%) (Fig. 6h–i). Enumeration of total cells administered as ACT translated to approximately 1% of stem-like gp100-reactive TIL in their infusion products. These data comprehensively indicated that clinical responses observed after Rx3+vaccination were not because of significantly altered clonotypes or phenotypes between the different infusion products.

Finally, we evaluated the impact of concurrent vaccination on *in vivo* expansion and persistence of adoptively transferred TIL by deep sequencing of PBL TCR repertoire over 16 time points throughout the patient’s clinical course for over 3 years (Fig. 6k, **Extended Data Fig. 9c**). While total CD8 counts in PBL remained relatively stable with expected variations around lymphodepletion associated with each TIL-infusion, the immunodominant TCR-1 clonotype expressing CD8^+^ TIL underwent a dramatic expansion only after co-administration of concurrent vaccine (up to 100 cells/uL of blood following Rx3 and Rx4) while they were virtually undetectable after the first 2 TIL infusions (Rx1, Rx2) without vaccination (Fig. 6k, **Extended Data Fig. 9c**). TCR-7 clone was detected only once following Rx4 (after TIL+vaccine) indicating its subdominant low frequency in the infusion product. Collectively, these data suggest that at least in this patient, antitumor clinical responses coincided with the expansion and persistence of adoptively transferred tumor-specific TIL clones only after concurrent vaccination, and that the vaccine may have impacted the low frequency T_SL_ cells within largely differentiated, dysfunctional immunodominant gp100 TIL-clone within the infusion product corroborating the evidence from murine studies.

## Discussion

A paradox of T cell immunity in patients with cancer is that stem-like memory T cell phenotypic states with progenitor potential responsible for immunotherapy responses in ICB and ACT represent only a minority of the antitumor TIL compartment while the vast majority of these cells are in CD39^+^ dysfunctional, exhausted states^13,15,28,31,37,63–65^. As ACT is extended to new cancer indications, a deeper understanding of T cell phenotypic states will be essential for maximizing clinical benefit. Therapeutic vaccines as monotherapies have largely not been effective in treating patients with advanced metastatic cancer^66–68^. However, there is a renewed interest in combining therapeutic vaccines in combination with ICB and ACT. Previous studies showed that vaccines can induce *de novo* antitumor T cells with stem-like states^21,43,69,70^. Because prior murine ACT studies were generally accompanied by concurrent vaccination it was difficult to disentangle the impact of vaccination on the specific T cell phenotypic states in the infusion product^44,71,72^.

In this study, the neoantigen ACT model we developed enabled us to specifically investigate the impact of therapeutic cancer vaccines on an ineffective ACT composed largely of T_dys_ cells. We demonstrate that concurrent vaccination along with ACT reshaped the host tumor immune landscape. As early as day 3 after ACT, in the TME, vaccination resulted in increased proportion of T cells, pro-inflammatory APCs, along with a consistent reduction of *Chil3* anti-inflammatory TAM cells shown previously^43,69^ (Fig. 3). Because this remodeling of TME happened exclusively in all vaccinated groups, this is likely an independent vaccine-specific effect in enhancing ACT and does not appear to be specifically related to T_dys_-ACT rescue (Fig. 3f–3i).

Host antigen-presenting capacity was indispensable for vaccine-mediated augmentation of T_dys_-ACT since the effect was abolished in β2m-deficient mice, and upon blocking the B7.1/B7.2 costimulation pathways, which have been demonstrated in PD-1 mediated reinvigoration of antitumor immune response^56–58^. In contrast, ACT using CD39^lo^ T_SL_ cells (without vaccination) still retained antitumor response even in the context of impaired host antigen presentation (Fig. 1, Fig. 2, Fig. 3j–k). These data suggest that when ACT is administered with large numbers of phenotypically “fit” T_SL_ cells, vaccine-mediated augmentation might not be necessary, since these cells already have the ability to differentiate into antitumor effectors and mediate tumor regression even in the absence of vaccination^15,23,44,71,72^. Nevertheless, in the scenario of human TIL in metastatic cancers where cells have been exposed to chronic antigen for presumably years in some cancer types, such T_SL_ are expected to be a minority of total antitumor T cell fraction in the infusion^15^ which need to be re-amplified using antigen-specific vaccines.

Multiple lines of evidence in our study indicated that systemic concurrent vaccination had a major impact on the quantity and quality of ineffective T_dys_-ACT, likely by amplifying on the relatively low frequency of T_SL_ within T_dys_-ACT. First, stem-like T cells within the murine and human TIL-ACT infusion products were generally represented at < 5% of T cells (Fig. 5a–c, Fig. 6f–i). Second, we detected significant expansion of CD39^−^ T_SL_ in host tumor and lymphoid organs after adoptive transfer of terminally differentiated CD39^+^CD69^+^ T_dys_-cells which we showed previously have minimal self-renewal potential^15^. Third, vaccination mediated tumor regression in mice treated with ACT using small numbers of CD39^lo^ T_SL_ (as low as 1000 cells per mouse) even as these cells were unable to mediate tumor regression by themselves (Fig. 5g–i). Fourth, congenically marker-based tracking of “spiked-in” CD39^lo^ T_SL_ within CD39^+^CD69^+^ T_dys_-ACT indicated a consistent amplification of CD39^lo^ T_SL_ from as low as 0.1% frequencies (Fig. 5j).

Although the data presented here are reminiscent of proliferation of Tcf-1^+^ “progenitor-exhausted” T_SL_ cells in response to ICB in murine models, in our experiments anti-PD-1 administration was insufficient to rescue T_dys_-ACT (Fig. 1b)^19–22^. In ICB models, anti-PD-1 treatment promotes the differentiation of antitumor T_SL_ cells from either the lymph node or within the tumor into transitional effector and terminally differentiated T cells to generate antitumor response^73–76^. Vaccination combined with ICB also can have a similar synergistic antitumor effect^42,77^. However, in these studies, it was unclear whether vaccination induced new antitumor T cell clones, or whether providing the “right context” of stimulation on APCs had the ability to rescue or de-differentiate pre-existing dysfunctional T cell clones^45^. In our studies, we observed minimal *de novo* vaccine induced immunity in murine and human TIL-ACT due to lymphodepleted hosts but dramatic expansion of low-frequency T_SL_ cells from the ACT infusion product. Thus, we conclude that amplification of rare T_SL_ cells likely explains synergistic antitumor effects of vaccination when combined with either ICB or ACT.

Extensive analyses of adoptively transferred human TIL in a melanoma patient further support the data that vaccines amplify low-frequency T_SL_ clones (Fig. 6). First, the high clonotypic concordance within gp100-specific T cells between the TIL-infusion products which consist of one clear immunodominant gp100-clone and no newly identified GP100-clones post-vaccine rules out de novo vaccine-induced T cell response. Second, the high phenotypic concordance within the gp100-TIL clones indicates that the vaccine enhanced the antitumor function of phenotypically dysfunctional ineffective TIL infusions (Rx1, Rx2) into TIL that mediated clinical antitumor response (Rx3, Rx4). Importantly, when analyzed in context of TIL-ACT infusions administered to our prior retrospective cohort of melanoma patients^15^ (Fig. 6g), it is clear that the frequency of administered gp100-TIL and stem-like gp100 TIL was low in the infusion product (~1% of all cells) and comparable to those of nonresponders, indicating that they were unlikely to have mediated antitumor responses by themselves. Thus, from a translational perspective, combining therapeutic vaccination with ACT containing a minor population of T_SL_ cells represents a feasible strategy to amplify and sustain antitumor immunity when most TIL are dysfunctional.

In conclusion, our work establishes that vaccination acts as a physiological amplifier of the rare but critical stem-like T cell populations that underpin durable antitumor responses. These insights open new avenues for next-generation immunotherapies that combine precision vaccination with personalized adoptive cell transfer, offering a rational path toward more consistent and long-lasting cancer remissions.

### Limitations of this study:

In this study, we have not explored whether the myeloid immune subsets found in the TME have antigen presentation function in the tumor or elsewhere to modulate ACT during vaccination. While we demonstrate through multiple analyses the effect of vaccination on the amplification of low frequency T_SL_ in the ACT product, we cannot completely rule out a second source of *TCF7+* cells that may arise from the re-expression of a subset of T_dys_ cells suggested in some studies^78^. Finally, our extensive clonal and phenotype tracking of human TIL clones during ACT with vaccination experience is limited to one patient, and data from other TIL-ACT patients treated with concurrent vaccines are necessary to generalize the findings from this study.

## Methods

### Cell Lines.

B16^KVP^, a B16F10 (B16) murine melanoma line engineered to overexpress a modified gp100 at positions 25–27 (**EGS**RNQDWL→**KVP**RNQDWL) to a high affinity neoepitope restricted by H-2Db was utilized as described previously^15,49^. B16F10 (ATCC); HPAC (ATCC); SK23 (ATCC) were used according to ATCC instructions. MC38 and other human melanoma lines (624 and 660) were obtained from Surgery Branch, NCI or NIH collection. For epithelial tumor models, MC38 colon cancer cell lines engineered to express gp100^KVP^ neoepitope were used (MC38^KVP^) as additional tumor models for ACT. For murine TIL experiments, the original B16F10 tumor line was used to implant into Pmel TCR-transgenic mice to grow tumors. Tumor cells were cultured in D10 medium (RPM1 supplemented with 10% fetal bovine serum [FBS; Hyclone], 1% penicillin-streptomycin (Life Technologies), HEPES, and 0.1% 2-mercaptoethanol (Life Technologies). For human tumor lines, the same tumor cell media without 2-mercaptoethanol was used. Cells were harvested by removing media, washing with 1x PBS (Gibco), incubating with TryplE (Gibco), neutralizing with media and centrifugation. Cells were passaged as needed for experiments, and early passage aliquots were used for any functional studies. Each vial of B16, B16-KVP, MC38, and MC38-KVP were tested upon thawing for Pmel T cell recognition to authenticate the presence or absence of neoepitope before further studies.

### Murine models and Adoptive cell transfer experiments

#### Mice.

Animal studies were performed under guidelines set by NIH Institutional Animal Care and Use Committee (IACUC# SB-126–5-A), Animal Research Advisory Committee (ARAC) and the NCI-Bethesda Animal Care and Use Committee. 4–5-week-old C57BL/6NCr mice were purchased from Charles River Laboratories. B6.129P2-B2mtm1Unc/DcrJ (β2m^−/−^ knockout) mice were purchased from Jackson Laboratories. Pmel-1 TCR transgenic mice were developed in Surgery Branch, NCI and crossed to B6.PL-Thy1a/CyJ (Thy1.1, Jackson Laboratories). B6 (carrying the hgp100-specific rearranged TCR transgene (Vβ13)) were purchased from Jackson Laboratories. All mice including Thy1.1^+^Thy1.2^+^ or Thy1.1^+^Thy1.2^−^ backgrounds were bred at the NIH Animal care facility.

#### Culture of Pmel T Cells and TIL.

Spleens from Pmel TCR-transgenic mice were mechanically dissociated to release splenocytes, which were then passed through a 70-μm cell strainer using cold, sterile complete RPMI 1640 medium (supplemented with 2μM glutamine, 100 U /ml penicillin and streptomycin, and 10% FBS). Cells were centrifuged at 1,200 rpm for 5 minutes, and red blood cells were lysed using ACK lysis buffer for 5 minutes on ice. Following lysis, splenocytes were washed twice with sterile complete RPMI 1640 medium and were cultured in murine T cell media composed of RPMI-1640 medium supplemented with 10% FBS (Hyclone), 2 mM L-glutamine, 1% penicillin-streptomycin, 1% MEM non-essential amino acids, 1% sodium pyruvate, and 0.1% 2-mercaptoethanol (Life Technologies). Splenocytes were stimulated with 1 g/mL hgp100^KVP^ peptide and 60 IU/mL recombinant human IL-2 (Aldesleukin). Secondary repeat stimulation for further differentiation on day 5 was performed using plate-bound anti-CD3ε (1μg/mL) and anti-CD28 (1μg/mL) antibodies (eBioscience). After 10 days in culture the cells were FACS-sorted into less dysfunctional CD39^lo^ T_SL_ and CD39^+^CD69^+^ T_dys_ cells for tumor treatment experiments. For murine TIL-ACT experiments, TIL was extracted from B16F10 tumors implanted in Pmel TCR-transgenic mice using the procedure described in the below section. Extracted TIL were expanded in presence of peptide-pulsed (1μg/mL human gp100^KVP)^, irradiated bone-marrow derived dendritic cells generated 5–7 days prior to enabling gp100^KVP^-specific TIL extravasation and outgrowth. Pmel TIL were grown for 7–10 days in T cell media supplemented with 60 IU IL2 and 0.5 μg/mL human gp100^KVP^ peptide before use for immunophenotyping analysis or for ACT experiments.

#### Murine tumor processing and TIL Analysis.

For TIL extraction, all tumors were excised, minced, and enzymatically digested using Mouse Tumor Dissociation Kit (Miltenyi Biotec) and gentleMACS Octo Dissociator (Miltenyi Biotec) as per manufacturers’ protocol. The digested tissue was filtered through 100 μm and 40 μm nylon mesh to obtain single-cell suspensions, which were then subjected to ACK lysis for 5 minutes on ice and washed in 1X PBS containing 2 mM EDTA. To block Fc receptors, cells were incubated with Fc-block (2.4G2, BD Biosciences) for 30 minutes on ice prior to staining. T cells were identified by flow cytometry using markers for CD3ε (BD Pharmingen, clone 145–2C11), CD4 (Biolegend, clone GK1.5), CD8α (Biolegend, clone- 53–6.7), and congenic markers Thy1.1(Biolegend, Clone OX-7) and Thy1.2 (Biolegend, Clone 53–2.1). T cells were identified by flow cytometry using markers for CD3ε, CD4, CD8α, congenic markers Thy1.1 and Thy1.2, Vβ13, and T cell phenotypic markers 4–1BB, CD39, CD69, PD-1, TIM-3 (described below).

#### Adoptive Cell Transfer Immunotherapy.

For ACT immunotherapy experiments, female C56/BL6 mice aged 6–8 weeks were injected with 2 × 10^5^ of B16^KVP^ or MC38^KVP^ tumor cells. Tumor growth and survival were monitored until mice were reported sick or tumor sizes reached the limit threshold per the IRB guidelines. Tumor bearing mice were randomized and sub-lethally irradiated (600 cGy) the day prior to cell transfer. Data from mice that had treatment unrelated deaths before or during ACT administration were excluded. No other data were excluded in murine treatment experiments. For murine TIL experiments, Pmel TCR-transgenic mice were implanted with the original B16F10 tumor line for 10–11 days, and then tumors were harvested to grow Pmel TIL *in vitro* as detailed in the section above before ACT. On the day of ACT, Pmel-1 CD8^+^ T cells grown *in vitro* derived from Pmel TCR-transgenic mice were FACS-sorted either as bulk, for specific T cell subsets of interest. In most ACT experiments, FACS-sorted CD39^lo^ T_SL_ and CD39^+^ CD69^+^ T_dys_ cells were used for ACT. In some experiments, additional subsets of CD39-expressing T cells (CD39^lo^, CD39^med^, CD39^hi^), or PD1^+^ TIM3^+^ T_dys_ cells were utilized for ACT. In most experiments, unless indicated, recipient mice (n = 5 per group) received intravenous tail vein injections of 0.75 × 10^6^ or 1 × 10^6^ bulk or FACS-sorted Pmel-1 CD8^+^ T cells or TIL, followed by three daily intraperitoneal injection of 3.6 × 10^5^ IU Proleukin (Clinigen). In some experiments 100ug of agonistic anti-mouse CD40 (FGK4.5/FGK45, BioXcell) or anti-PD1 antibody (RMP1–14, BioXcell) were administered intraperitoneally along with ACT. In mice that received vaccinations, 2e7pfu of recombinant VACV^KVP^, or 2e8 pfu Ad^KVP^, or 5ug of KVPRNQDWL neopeptide and 100ug of agonistic anti-mouse CD40 (FGK4.5/FGK45, BioXcell) were intravenously injected along with T cells. Tumor area was calculated as length × width. Mice with tumors approaching 2cm in diameter were euthanized. Results are presented as mean ± SEM. Tumor measurements for cell therapy experiments were performed in a double-blinded randomized manner, coded by the investigator and by the personnel administering the cell therapy by separately de-identifying the animals. An independent member unblinded and reconciled the two codes at the completion of the experiment.

#### In vivo tracking of adoptively transferred T_SL_ and T_dys_ cells in mice.

Congenically marked Pmel T cells were derived from splenocytes isolated from Thy1.1^+^ Thy1.2^−^ or Thy1.1 ^+^Thy1.2^+^ mice. T cells from these two groups were separately expanded *ex viv*o for 11 days with repeated stimulation as described above. On the day of adoptive transfer, congenically marked T cells were FACS-sorted for CD39^lo^ T_SL_ (from Thy1.1^+^Thy1.2^+^ mice) and CD39^+^ CD69^+^ T_dys_ cells (from Thy1.1^+^Thy1.2^−^ mice) and mixed at indicated ratios and 1 × 10^6^ total T cells were intravenously injected into recipient mice, which were then challenged with 2 × 10^7^ PFU VACV^KVP^. Mice received intraperitoneal IL-2 (3.6 × 10^5^ IU in 0.5mL PBS) once daily for three consecutive days. Splenocytes from host mice harboring the mixed transferred populations were harvested and used for counting absolute numbers of adoptively transferred T_SL_ and T_dys_ cells.

### Flow Cytometry Analysis and Cell Sorting

Single-cell suspensions of *in vitro* expanded murine T cells, or adoptively transferred murine TIL, human TIL infusion products, human PBL-derived T cells, and post-ACT human T cells were washed and resuspended in FACS buffer (PBS, 2% FBS) prior to staining. For FACS-sorting sterile-filtered fresh FACS-buffer was used. For flow cytometric cell acquisition Fortessa or Symphony flow cytometer was used. For FACS-based cell sorting, Sony two-way SH800 or four-way MA900 instrument was used. The following murine T cell antibodies were used: CD3ε (BD Pharmingen, clone 145–2C11), CD4 (Biolegend, clone GK1.5), CD8α (Biolegend, clone- 53–6.7), congenic markers Thy1.1 (Biolegend, Clone OX-7), Thy1.2 (Biolegend, Clone 53–2.1), Vβ13 (Biolegend, Clone MR12–4), CD39 (Biolegend, Clone Duha39), CD69 (Biolegend, Clone H1.2F3), PD1 (Biolegend, Clone RMP1–30), TIM3 (Biolegend, Clone RMT3–23), 41BB (Biolegend, Clone 17B5). The following human antibodies were used for human TIL immunophenotyping: CD8 (BD Pharmingen, Clone RPA-T8), CD4 (BD Pharmingen, Clone: SK3), CD39 (Biolegend, Clone A1), CD69 (BD Pharmingen, Clone FN50), PD1 (Biolegend, Clone: EH12.2H7). TCR-engineered human T cells were detected by APC or PE Hamster Anti-Mouse TCR β Chain (Catalog 561081 or 553174, BD Biosciences) after gating on T cells. Cell expansion was monitored by enumerating live CD8^+^ T cells using a FACS-based CountBright bead assay (Life Technologies). Data was analyzed using FACSDiva Software (v8.0.01), Sony SH800 or FlowJo software (FlowJo v10.8.1 (BD)) and subsequent statistical analyses were performed using Graphpad v10 or R v4.4.2. Gating strategy is outlined in Extended Data Fig. 2.

### Human Melanoma patient TIL-studies

#### TIL-ACT for melanoma patient with and without vaccination

Clinical course and ACT treatments administered to a patient with metastatic melanoma along with vaccination under NIH Institutional Review Board (IRB)-approved clinical protocol, NIH Office of Intramural Research, NIH Deputy Director for Intramural Research (DDIR). Clinical materials and samples were obtained from completed NCI clinical protocol 04-C-01552, and 03-C-0277, NCT00001823, 99-C-0128, under informed consent was previously described^62^. All donors in the protocol provided written informed consent in accordance with the Declaration of Helsinki. Briefly, cervical lymph node tumors were resected, sectioned into individual fragments and cultured in single wells of 24-well plates containing media supplemented with interleukin-2 (IL-2) as previously described^62,79^. TILs were cultured in 300 IU/mL IL-2 in a 1:1 mixture of complete human media (comprising 10% human AB serum, 100 U/mL penicillin, 100μg/mL streptomycin, 2 mM L-glutamine, and 12.5 mM HEPES) and AIM-V medium. The lymphocytes were cultured for three weeks until sufficient numbers were available for tumor antigen reactivity testing. Aliquots were cryopreserved or further expanded for therapeutic use. Reactive lymphocytes underwent a rapid expansion protocol (REP) involving soluble OKT3 (anti-CD3) antibody (Ortho Biotech, Bridgewater, NJ) and IL-2 in the presence of irradiated peripheral blood mononuclear cells. This REP was conducted over a 14-day period, resulting in greater than 1000-fold expansion of the lymphocytes. The patient received two infusions of autologous TILs (Rx1: administered intravenous and Rx2: administered intraarterial). Third TIL infusion (Rx3) was obtained from same TIL source as Rx1 and Rx2, while for the fourth TIL infusion (Rx4), an additional gp100-reactive fragment was added to the TIL starting cultures. Approximately 2–4 hours prior to TIL-ACT with Rx3 and Rx4, a fowlpox vaccine was administered intravenously encoding the gp100: 209–217 minigene with higher affinity 210M modification at the threonine at position 2 (referred to as GP209M). A second boost of the vaccine was administered on day 28 without lymphodepletion but along with intravenous high dose aldesleukin injections. For additional clinical details including tumor responses see ref 62.

#### Tetramer-based detection of GP100-reactive TIL from Human TIL infusion.

Human gp100-reactive TILs from patient infusion products and peripheral blood were identified by tetramer staining. HLA-A*02:01-GP209M-specific tetramers were generated using UV-exchangeable biotinylated monomers (NIH Tetramer Core Facility, Atlanta). Briefly, 10ug of biotinylated HLA-A*02:01 monomer containing UV-labile peptide (NIH Tetramer Core, Emory University) was co-incubated with 40ug GP209M peptide (IMDQVPFSV) in 100uL 1x PBS. UV-exchange was performed for 90 minutes on ice. Following UV-exchange, 0.8ug each of Streptavidin-APC (Catalog 405207, Biolegend) and Streptavidin-PE (Catalog 405203, Biolegend) was added in four 15-minute intervals until saturation. At the end of tetramerization, final volume was made up to 200uL with 1x PBS with 2% BSA and stored in 4C until use. For staining, cells were washed, stained with dual-colored tetramers (1:50 dilution, 30 min, 4°C), and then stained with surface antibodies for CD3, CD4, CD8, CD39, CD69 or other cell surface antibodies for an additional 20 minutes on ice in the indicated antibody volumes above. Propidium iodide or DAPI was used for dead cell exclusion. Flow cytometry gates were set using fluorescence minus one (FMO) controls. Tetramer-stained TIL infusions or PBL were FACS-sorted into single cell TCR-sequencing plates.

#### gp100-reactive TCR isolation, reconstruction and testing.

Total RNA was isolated using Norgen’s Total RNA Purification Plus Kit. TCR-seq libraries were generated following the manufacturer’s protocol for Takara’s SMARTer Human TCR a/b Profiling Kit v1. Initial data processing was performed on FASTQ files using MixCR^80^ with settings optimized for the SMARTer Human TCR a/b kit. TCR diversity across samples was assessed using the R package immunarch^81^, which calculates the effective number of types to account for both clone count and relative abundance. Candidate TCRs with TRBV-CDR3β-TRBJ, and TRAV-CDR3α-TRAJ were fused to modified murine TRBC and TRAC chains, respectively. TRB and TRA were synthesized as a single-chain separated by a furin SGSG P2A linker and cloned into pMSGV-1 (GenScript, Piscataway NJ). Retroviral supernatants were produced using HEK-293GP packaging line, as previously described^82^. Briefly, 0.7–1×10^6^ cells per well were plated in 6-wells poly-D-Lysine-coated plates and co-transfected with 2μg/well TCR-encoding p-MSGV-1 and 1.4μg/well envelope-encoding pRD114 plasmids using Lipofectamine 2000 (Invitrogen, Cat. 11668–019), and supernatants were collected 48–72hrs following transfection. Next, supernatants were plated in non-tissue culture treated-plates pre-coated with 10–20 μg/mL retronectin (Takara, T100B) and centrifuged at 2000xg, 32°C for 2hrs. Subsequently, supernatants were discarded and 1–2×10^6^ stimulated healthy donor PBLs (0.5×10^6^ cells/mL) were centrifuged onto the retrovirus-coated plated 350xg for 10min. Transduced cells were removed and transferred into tissue-treated plates after 24 hours and grown in rhIL-2-containing media for 10 days before screening. TCR-engineered human T cells were detected by APC or PE Hamster Anti-Mouse TCR β Chain or via tetramer after gating on T cells. Confirmed TCRs were used for scRNA and in vivo persistence studies (**TCR-1**: CDR3a-CATVADRDDKIIF; CDR3b-CASSFGRGQPGYTF and **TCR-7**: CDR3a-CAVSLIQGAQKLVF, CDR3b-CASSPGGNEQFF).

#### Sample Preparation for ImmunoSeq TCR-Vβ Deep Sequencing.

Bulk T cells from peripheral blood or TILs (pre- and post-ACT) were FACS-sorted for live CD8^+^ T cells, pelleted, and snap-frozen. TCR-Vβ deep sequencing was performed by Adaptive Biotechnologies (Seattle). Data were analyzed using the ImmunoSeq Analyzer, and only productive TCR rearrangements were included in frequency calculations.

#### Calculation of circulating CD8^+^ and CD8^+^ gp100-specific T cells in TIL+vaccine ACT patient.

Absolute peripheral blood cell counts were obtained from standard CLIA-certified laboratory assays utilized at the NIH Clinical Center. Count values were integrated with CD8 and tetramer stain flow cytometry results of research blood samples in order to calculate the total number of CD8^+^ and CD8^+^ gp100-reactive T cells at various treatment time points after receipt of ACT. All inferred absolute post-ACT PBL counts were obtained at time points were +/− 2 days within the CDR3b survey sequencing data. Total CD8 number (cells/μL) was directly obtained from the CLIA-certified TBNK (T cell B cell NK cell) flow cytometry assay utilized at the NIH Clinical Center (“Total CD8”, Fig. 6k). Absolute lymphocyte counts obtained were used to estimate the total number of CD8 T cells (cells/μL) and the proportion (%) of CD8+Tetramer+ T cells, as informed by flow cytometry, was then used to estimate of the number of gp100-reactive CD8 T cells at that time point (“GP209M TCR-1”,“GP209M TCR-7”, Fig. 6k).

### Murine T cell and human gp100 TCR functional assays

For Pmel serial tumor killing assay, cells were co-cultured on RFP-expressing B16KVP tumor lines for a period of 72 hours at 5:1 E:T ratios (10,000:2000) in T cell media without any cytokines, in repeats of four wells per condition using the IncuCyte S3 live cell analysis machine (Sartorius) with measurement of red object count fluorescence every 4 hours. Tumor cells were plated first onto 96-well flat-bottom plates and allowed to adhere over 1 hour prior to adding T cells. Tumor killing curves were generated by normalizing each time point to the initial starting measurement of tumor fluorescence object count total area. Human TCR-transduced T cells were evaluated for functional reactivity via overnight co-culture. Transduced T cells were incubated overnight within ELISpot plates (Millipore) along with patient derived antigen presenting cells pulsed with hgp100^KVP^-peptides (1μg/mL and lower dilutions). For overnight tumor cell coculture of TCR-transduced T cells with melanoma and non-melanoma tumor lines, 1×10^5^ cells were cocultured with 5×10^4^ tumor cells in T cell media without any cytokines in an ELISpot plate. Following overnight co-culture, cells were stained with fluorescently labeled antibodies (CD3, CD8, CD4, CD137, murine TCRβ) (BD Biosciences). ELISpot plates underwent development to detect interferon gamma (IFN-γ) release while fluorescently stained T cells were analyzed via flow cytometry for upregulation of CD137 (4–1BB). Positive results were determined by IFN-γ release or CD137 upregulation >= twice the background as determined by control wells containing T cells, APCs and DMSO alone. Flow cytometry data was analyzed via FlowJo software (FlowJo v10.8.1 (BD)).

### Single cell transcriptomics (scRNA-seq) analysis

#### Sample Preparation for murine and human single cell transcriptomic analysis.

Murine CD45^+^ cells or murine Pmel T cells were stained with relevant antibodies in FACS buffer. For murine CD45+ immune landscape analysis, the following cell surface DNA-barcoded hashing antibodies (Biolegend) and Thy1.1 antibody (TotalSeq-B0060, clone 5E10, Biolegend) were co-stained to facilitate CITE-Seq analysis by 3’ sequencing with the following groups: TotalSeq-B0302 (untreated), TotalSeq-B0307 (VACV^KVP^ only), TotalSeq-B0310 (CD39^+^CD69^+^ T_dys_ only), and TotalSeq-B0306 (CD39^+^CD69^+^ T_dys_ + VACV^KVP^). Approximately ten thousand CD45+ cells were FACS-sorted from each of the 4 groups into a single eppendorf tube at a final cell concentration of 1e6/mL cells in the cell sorting buffer (50% 1x PBS with 50% FBS) for scRNA capture. For murine Pmel T cell infusion products (CD39^lo^ T_SL_ or CD39^+^CD69^+^ T_dys_ cells) the two groups were separately captured without hashing for transcriptome only scRNA analysis. For human TIL infusion scRNA, tetramer-stained cells were washed twice to remove unbound antibodies and FACS-sorted (Sony SH800 two-way sorter), and subject to 5’ single cell transcriptomic (5’ scRNA) sequencing and TCR-sequencing using Next-GEM v2 kit (10X Genomics) for Rx1 infusion product and Rx3 infusion products and captured, sequenced separately.

#### *scRNA-seq data processing and analysis*.

Murine immune cells and human TIL single-cell RNA-sequencing (scRNA-seq) libraries were processed using Cell Ranger (10x Genomics) software. For human TIL transcriptomic analyses, 5’ scRNA-seq libraries were aligned to GRCh38–2020-A and vdj_IMGT_human for TCR repertoire analysis using cellranger version 6.1.1 version. For murine TIL and post-ACT TME analyses, 3’ scRNA-seq libraries were aligned to mm10–2020-A using cellranger version 7.0.1 for gene expression analysis. Raw sequencing files were demultiplexed using the cellranger mkfastq function. All sequencing reads were aligned to hg38 reference genome or mm10 reference genome (for murine cells) along with custom tetramer barcodes, VDJ sequencing was performed to identify TCR-sequences per cell. Gene expression and V(D)J feature matrices were generated using the cellranger count and cellranger vdj pipelines, respectively, under default parameters. Output HDF5 files were imported into Seurat (v4.0) framework (https://github.com/satijalab/seurat) for downstream gene expression analysis. Cells were retained for downstream analysis if they met the following quality control criteria: ≥250 detected genes per cell, mitochondrial RNA content <20%, and ≥500 unique molecular identifiers (UMIs) per cell. Genes with low expression (total UMI count <4 across all cells) were excluded. For murine scRNAseq CD45+ immune cell profiling experiments, tumor cell impurity were excluded before reclustering as indicated in Extended Data fig. 6. For demultiplexing hashed sample analysis, cells that failed thresholds to be called a clear “hash-call” (i.e. one of the 4 groups) were excluded from further analyses. For human and murine scRNAseq/scTCR-seq data, cells with low number of detected transcripts (total UMI <4 across all cells) and high mitochondrial cell content, and doublet cells were excluded as established as standards in the field by prior studies^16,43,69^. For murine Pmel T cell infusion product analysis, CD39^lo^ T_SL_ or CD39^+^CD69^+^ T_dys_ cells sequenced separately were integrated to remove any possible batch effects and then analyzed for transcriptomic differences. For human TIL-ACT scRNA analysis, only cells that had detectable CD8 transcripts were used for TIL infusion phenotypic analysis. During phenotypic clustering analyses, TCR variable (TR[AB]V) and joining (TR[AB]J) genes were excluded to minimize noise and reduce bias introduced by TCR gene-usage biased clusters.

scRNA-seq visualizations and analyses were performed after UMAP construction. To demultiplex hashed murine CD45+ immune cells from TME landscape analysis, hash reads were normalized and z-transformed for each cell barcode to facilitate a hash call that corresponded to the hash value for each cell barcode. The “Featureplot” function from Seurat was further used to confirm hash call specificity for each experimental group (untreated, VACV^KVP^ only, T_dys_ only, T_dys_ + VACV^KVP^). Cluster distributions were calculated for each group after normalizing for each hashed group. Cluster distributions were calculated for both murine and human immune cells. For human gp100 TIL cluster distributions the TCRB expressing cells were subset based on barcode identity and then further analyzed. Differentially expressed genes were analyzed by using the “cluster.markers” function of Seurat and visualized by heatmaps after averaging per each cluster or by performing pseudobulk analysis for populations of interest. Only differentially expressed genes with adjusted P-values < 0.05 were considered for analysis.

### Single cell gene signature (scGSEA) analysis

Each scRNA-Seq library of murine T cell infusion products or human TIL infusion products was checked for quality and assembled into a single Seurat object. Single cell gene set enrichment analysis (scGSEA) was performed on the single murine T cells or human TIL for stem-like, dysfunctional, and other published gene-signatures using AUCell R package or using the “AddmoduleScore” function in Seurat. Top decile gene signature expressing cells were visualized on the umap plots using the Featureplot function, or the entire clusters median gene signature scores were analyzed to identify the best fit cluster for each gene signature.

### Quantification and Statistical Analysis

Data was analyzed using FlowJo v10.8.1 (BD) for Flow Cytometry, Immunoseq Analyzer (Adaptive Biotechnologies) for human bulk TCR sequencing. For single cell RNA and TCR sequencing Seurat (v4.0) (https://github.com/satijalab/seurat) was used using R statistical environment v4.4.2. For oher statistical analyses: R v4.4.2, Graphpad Prism 10 and MS Excel were used. Sample sizes were determined based on prior experience with similar experiments^44,49^. Samples with technical failure were excluded. All *in vivo* experiments were conducted with n=5–6 mice per group. Statistical analysis for *in vivo* experiments was performed by One-way ANOVA with Tukey’s multiple comparisons test unless indicated. Statistical analyses were performed using two-tailed Mann-Whitney U tests for nonparametric data and paired t-tests for paired analyses. For ACT cell quantification analyses, data points from each mice (n = 6) or fold-change were calculated relative to counterpart unvaccinated groups from spleen, DLN, and tumor. Statistical significance by unpaired non-parametric Mann-Whitney test. For multiple comparisons, ANOVA with Bonferroni correction was used. P values ≤ 0.05 were considered significant. Data are presented as mean ± SEM unless otherwise indicated. Statistical analyses were performed using Prism (GraphPad Software). For differentially expressed gene analyses, false discovery rate of 0.05 was assumed with adjusted P-values after multiple corrections being the threshold for further analysis.

## Supplementary Material

**Supplementary Table 1.** Differentially expressed genes (DEG) per cluster in scRNA-seq analysis of CD45+ immune cells in the tumor microenvironment at 3 days post-ACT. Genes with adjusted p-values < 0.05 are shown.

**Supplementary Table 2.** Differentially expressed genes in scRNA-seq analysis of Pmel infusion products derived from CD39^lo^ T_SL_ cells and CD39^+^CD69^+^ T_dys_ cells. First tab shows pseudobulk analysis comparing all cells from CD39^lo^ T_SL_ cells and CD39^+^CD69^+^ T_dys_ cells. Second tab shows per cluster DEGs with adjusted p-values < 0.05 are shown.

**Supplementary Table 3.** Differentially expressed genes per cluster in scRNA-seq analysis of TIL infusion products administered to metastatic melanoma patient integrating both first infusion (Rx1) and TIL infusion administered along with concurrent GP100-vaccination (Rx3). DEGs with adjusted p-values < 0.05 are shown.

Supplementary Files

This is a list of supplementary files associated with this preprint. Click to download.

- SupplementaryTable3.xlsx
- SupplementaryTable1.xlsx
- ExtendedFiguresv3020526.docx
- SupplementaryTable2.xlsx

## Data Availability

This study did not generate new unique reagents.

## Data and Code Availability

The data that support the findings of this study are available from the corresponding author upon reasonable request. Single cell transcriptomic sequencing analyses from murine experiments and single cell count matrices from human TIL can be accessed at Figshare on https://figshare.com/projects/_b_Impact_of_concurrent_vaccination_on_dysfunctional_T_cell_therapy_against_cancer_b_/272587. Deidentified Fastq files corresponding to scRNA sequencing and TCR-sequencing datasets from human TIL generated in this study are uploaded on the PII-protected controlled-access database of Genotypes and Phenotypes (dbGaP) (submission in progress). No unique code was generated for this study. All other data supporting the findings of this study are included within the published article.
